# The Emerging Role of METTL3 in Lung Diseases

**DOI:** 10.3390/ijms27010085

**Published:** 2025-12-21

**Authors:** Yishu Dong, Ying Liu, David Marciano, Adel Nefzi, Stephen M. Black, Ting Wang

**Affiliations:** 1 Center of Translational Science, Florida International University, Port St. Lucie, FL 34990, USA; 2 Department of Cellular and Molecular Medicine, Herbert Wertheim College of Medicine, Florida International University, Miami, FL 33199, USA; 3 Department of Environmental Health Sciences, Robert Stempel College of Public Health and Social Work, Florida International University, Miami, FL 33199, USA

**Keywords:** METTL3, N6-methyladenosine (m^6^A), lung disease, ALI/ARDS

## Abstract

N6-methyladenosine (m^6^A) represents the most abundant and tightly controlled modification within eukaryotic mRNA, critically influencing RNA metabolism and function. The m^6^A methyltransferase Like-3 (METTL3), responsible for the complex’s catalytic function, has emerged as a central epitranscriptomic regulator governing mRNA stability, alternative splicing, nuclear export, and the efficiency of mRNA translation. Converging research shows that METTL3 is involved in the pathogenesis of numerous disorders via m^6^A-dependent, post-transcriptional regulation of gene programs controlling cell growth, migration, and immune pathways. Regarding pulmonary pathophysiology, METTL3-mediated m^6^A is tied to disease initiation and progression in conditions such as asthma, chronic obstructive pulmonary disease (COPD), idiopathic pulmonary fibrosis (IPF), lung infections, acute respiratory distress syndrome (ARDS). This review summarizes the contemporary evidence for METTL3’s roles and regulatory network in diverse pulmonary pathologies. We further highlight emerging strategies for targeting METTL3 as a potential therapeutic approach, underscoring its promise as a novel epitranscriptomic target. Beyond inflammatory and fibrotic disorders, we also summarize emerging evidence linking METTL3 to lung cancer and briefly outline other respiratory conditions (e.g., ILD, bronchiectasis, and secondary pulmonary hypertension), highlighting common translational themes and remaining gaps. Further studies are required to clarify the disease-specific and context-dependent actions of METTL3 and to advance the clinical translation of m^6^A-based therapies.

## 1. METTL3 and Its Post-Transcriptional Regulation

### 1.1. Posttranscriptional Regulation of Gene Expression

The human transcriptome comprises a diverse array of RNA species, including protein-coding RNAs (mRNAs) and various classes of noncoding RNAs, including transfer (tRNA), ribosomal (rRNA), small nuclear (snRNA), micro- (miRNA), lncRNAs, and circRNAs [1,2]. Each RNA type performs specialized functions that collectively orchestrate the regulation of gene expression [3]. Beyond their sequence information, RNA molecules undergo extensive chemical modifications on both their nucleotide bases and ribose sugars, serving as core regulatory nodes for gene expression at the post-transcriptional stage.

These RNA modifications have been increasingly recognized as key contributors to tumorigenesis and disease progression, as they influence rewiring cellular programs spanning proliferation, lineage specification, metabolic remodeling, and stress responses. To date, researchers have identified over 170 types of RNA chemical modifications, among which the most studied include m^6^A, m1A [4], 5-methylcytidine (m5C) [5], N7-methylguanosine (m7G) [2], N4-acetylcysteine (ac4C) [6], 2′-O-methylation [7], pseudouridine [8], and adenosine-to-inosine editing [9]. These chemical modifications are known to regulate mRNA stability, splicing, editing, export, translation, and decay, as well as the biogenesis and function of non-coding RNAs [10,11,12,13,14]. As such, they function as major determinants of post-transcriptional gene regulation in response to developmental cues, environmental stress, and disease stimuli, thereby ensuring the fidelity of cellular homeostasis and protein synthesis [15].

### 1.2. m^6^A Modification and the Role of METTL3

**Figure 1:**
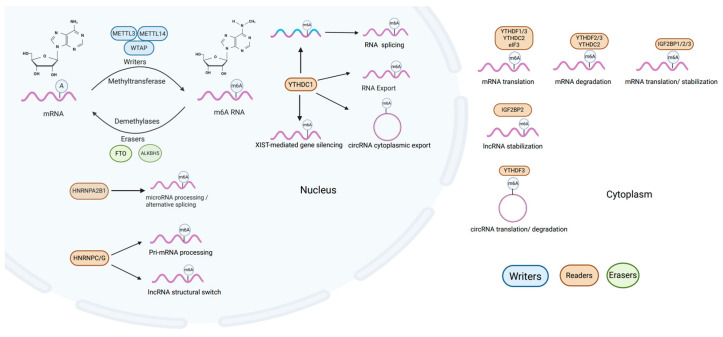
Cellular m^6^A modification processes. m^6^A modification involves “writers” like METTL3, METTL14 and WTAP that add methyl groups at the N6 position on coding RNA and non-coding RNA, influencing their processes and gene expression. This modification is reversible, with “erasers” such as FTO and ALKBH5 capable of removing methyl groups. “Readers” like YTH domain family readers, eIF3, HNRNPs, and IGF2BPs recognize and bind m^6^A-methylated RNA, thereby modulating splicing, nuclear export, degradation, or translation.

RNA m^6^A methylation stands as the predominant and most impactful form of post-transcriptional modification observed in both coding and non-coding RNA types [16,17]. It refers to the methylation of adenine (A) at the N6 position on RNA molecules, which plays a crucial role in RNA metabolism and function [18]. Just like DNA and histone methylation modifications, m^6^A modification on RNA is similarly introduced, removed, and identified by specialized enzymes known as writers, erasers, and readers (Figure 1).

m6A modifications are installed by “writers” (methyltransferases), primarily the METTL3-METTL14 complex together with WTAP [14,19]. This writer complex catalyzes methylation of adenosine at the N6 position, thereby shaping RNAmetabolism and ultimately influencing gene expression programs involved in development, differentiation, and disease. Interestingly, besides the core methyltransferases (METTL3-METTL14-WTAP) responsible for cellular m^6^A RNA methylation, some auxiliary and regulatory components of the m^6^A methyltransferase complex also exist, including VIRMA, which guides methylation to the 3′ untranslated region and near stop codons [20], ZC3H13, which anchors the writer complex to the nuclear matrix to support m^6^A deposition [21], and RBM15/RBM15B, which seem to preferentially target m^6^A methylation on long non-coding RNAs [22].

Compared to the complex members of writers, there are only two identified erasers (demethylases) catalyzing the demethylation of m^6^A to counteract writers; Fat mass and obesity gene (FTO) [23] and AlkB homolog 5 (ALKBH5) [24]. FTO and ALKBH5 are the two best characterized m6A demethylases (“erasers”) and have distinct expression patterns and subcellular localizations. FTO is broadly expressed in tissues such as the brain and adipose tissue, playing roles in metabolism, neurogenesis, and cancer. In contrast, ALKBH5 is primarily localized in the nucleus, and has more restricted expression, particularly in the testis and brain. Functionally, ALKBH5 is closely linked to RNA processing, fertility, and tumorigenesis.

Comparative context with other m^6^A regulators: in respiratory diseases, ALKBH5 and FTO oppose METTL3 by erasing m^6^A marks, frequently shaping metabolic adaptation and stress responses; YTH proteins (YTHDF1/2/3, YTHDC1/2) and IGF2BPs determine the fate of m^6^A-tagged transcripts (translation, decay and stabilization). Therapeutic entry points thus include writers (METTL3), erasers (ALKBH5/FTO), readers, or even site specific editing, each carrying distinct efficacy.

Readers are proteins capable of recognizing and binding to m^6^A-modified RNA. m^6^A modifications can be recognized by the YTH domain family readers, which comprise two subgroups: YTH domain family proteins 1–3 (YTHDF1–3) and YTH domain-containing proteins 1–2 (YTHDC1-2) [25,26,27]. In addition, several m6A readers have been reported, including eukaryotic translation initiation factor 3 (eIF3) [28], heterogeneous nuclear ribonucleoproteins (HNRNPs) [29,30], and insulin-like growth factor-2 (IGF2) mRNA-binding proteins (IGF2BPs) to affect both coding RNA and non-coding RNA fate [31]. These factors cooperate to precisely regulate the level and location of m^6^A modification, adapting cellular responses to external and internal signals. Evidence indicates that the m^6^A modification of RNA is critically involved in XIST-mediated transcriptional repression, with YTHDC1 affecting the efficiency of XIST in silencing genes on the X chromosome [32]. Additionally, it is known to bind m^6^A-modified Hepatitis B viral transcripts, aiding in circular RNA relocation from nuclear to cytoplasmic compartments [33]. HNRNPC/G target modifiable sites on lncRNAs, facilitating the regulation of their structural configurations. This structural modulation acts as a switch, significantly altering the RNA’s ability to bind to DNA [34,35].

Among m^6^A regulators, METTL3 stands out as the rate-limiting RNA methyltransferase and a key component of the writer complex [36]. By dictating both the efficiency and specificity of m^6^A deposition, it exerts transcriptome-wide effects on gene expression. Multiple post-translational modifications act on METTL3 to regulate m^6^A through distinct mechanisms. METTL3 is modified by phosphorylation as well as SUMO conjugation. ERK-driven phosphorylation of METTL3 (S43/S50/S525) and WTAP (S306/S341) facilitates USP5-dependent deubiquitination, thereby stabilizing METTL3. This stabilization promotes m^6^A modification while reducing transcript lifetime [37]. In contrast, SUMOylation dampens METTL3’s catalytic activity, leading to reduced global m^6^A levels [38]. METTL3’s role is context-specific, functioning as either an oncogenic driver or a tumor-suppressive factor depending on the cellular environment [39,40]. Research has shown its involvement in various cancers and diseases [41,42,43,44]. METTL3-mediated m^6^A modification regulates cancer stem cell characteristics, proliferation, invasion, and metastasis in cancer [45]. In the context of lung diseases, METTL3-mediated m^6^A modification regulates the expression of key genes involved in cellular proliferation, differentiation, inflammation [46], and cell death, highlighting its significant role in the initiation and progression of pulmonary pathology [28].

### 1.3. Cellular Regulation of METTL3

The regulation of METTL3, a central methyltransferase in the m^6^A modification machinery, is essential for maintaining physiological homeostasis. METTL3 expression and enzymatic activity are tightly controlled at multiple levels within the cell, including post-translational modifications, protein–protein interactions, and modulation by signaling pathways, to ensure precise and dynamic regulation of m^6^A methylation.

Post-translational modifications (PTMs) such as phosphorylation and ubiquitination significantly influence METTL3’s stability, binding affinity, and methyltransferase activity [37,47]. For instance, ERK-mediated phosphorylation of METTL3 at S43, S50, and S525, and of WTAP at S306 and S341, facilitates USP5-mediated deubiquitination, leading to increased METTL3 stability. Stabilized METTL3 further promotes mouse embryonic stem cell (mESC) differentiation, tumorigenesis, and mRNA decay, highlighting METTL3’s multifaceted regulatory functions [37].

METTL3 functions within a catalytic complex known as METTL3–METTL14–WTAP (MMW). Interactions among these components influence not only the localization and substrate specificity, but also the structural integrity of the complex [48]. Interestingly, METTL3 itself supports WTAP protein stability, as both knockdown and overexpression of METTL3 lead to increased WTAP levels [49]. However, WTAP depletion does not destabilize the METTL3 complex but does impair its nuclear localization, thereby affecting its function in methylation regulation [50,51]. In addition, METTL3 has been shown to interact with eIF3h, a translation initiation factor, to enhance the translational efficiency of oncogenic mRNAs, linking m^6^A regulation to protein synthesis control in disease contexts [52]. Cellular signaling pathways also regulate METTL3 expression and activity, responding to environmental cues such as growth signals, inflammatory stimuli, or oxidative stress. For example, METTL3 can be modulated by phosphorylation status changes within the PI3K/AKT pathway, which is essential for maintaining cell viability [53]. Dysregulation of these pathways under pathological conditions can lead to aberrant METTL3 activity, contributing to disease development and progression.

In the context of lung diseases, METTL3 functions as a converging point for multiple regulatory signals that modulate fibrosis, inflammation, and tumorigenesis. Its dynamic involvement in both homeostatic and pathological processes positions METTL3 as a promising target for m^6^A-based therapeutic interventions [54,55]. The dual capacity of METTL3 to act as a promoter or suppressor of disease-related pathways underscores the need for context-specific targeting strategies.

In summary, METTL3 is intricately regulated through a network of intracellular modifications, complex formation, and extracellular signaling responses. These regulatory layers determine its impact on post-transcriptional gene regulation and ultimately influence lung disease outcomes (see next section). Further research is needed to delineate how METTL3’s cellular regulation shapes its function in lung pathophysiology, and to evaluate the feasibility of pharmacological modulation (via inhibitors or activators) of METTL3 as a therapeutic approach for pulmonary disorders.

## 2. METTL3 in Airway Diseases

### 2.1. METTL3 in Asthma

Asthma is a heterogeneous chronic airway disease that can be classified into eosinophilic, neutrophilic, paucigranulocytic, or mixed granulocytic subtypes based on the dominant inflammatory cell types in the airways. Overall, current evidence supports a context-dependent dual role of METTL3 in asthma (predominantly protective in Th2-dominant allergic asthma, but potentially pathogenic in neutrophilic endotypes).

Current research demonstrates that METTL3 plays a protective role in allergic asthma by suppressing Th2 immune responses. Specifically, METTL3 mediates m^6^A methylation of SOX5 mRNA, leading to its downregulation, which in turn inhibits Th2 cell differentiation and IL-4 production [56]. Moreover, the METTL3/miR-192-5p/SCD1 signaling axis regulates lipid metabolism, thereby affecting T cell differentiation and contributing to the pathogenesis of asthma [57]. Clinical and experimental evidence consistently indicates that reduced METTL3 expression is associated with increased disease severity in Th2-dominant asthma, highlighting the potential of m^6^A-dependent METTL3 activity as a therapeutic target for allergic airway inflammation.

In contrast, neutrophilic asthma associated with severe disease, is characterized by excessive infiltration of neutrophils. Neutrophil extracellular traps (NETs), web-like structures released by neutrophils, are implicated in persistent inflammation and tissue injury in various lung diseases, including asthma, COPD, and cystic fibrosis [58]. Matrix metalloproteinases (MMPs), as components of Neutrophil Extracellular Traps (NETs), play a role in the pathogenesis of asthma. Inhibition of MMPs has been shown to attenuate the intensity of the pathological processes associated with asthma [59]. Pham et al. reported that IL-8 promotes neutrophil autophagy and enhances NET formation in severe asthma patients, further contributing to epithelial damage and disease progression.

Interestingly, emerging evidence indicates that NETs can promote METTL3 transcription mediated by H3K27ac by activating P300 [60], suggesting a potential feedback loop between neutrophilic inflammation and METTL3 expression. However, conflicting findings suggest that METTL3’s function in allergic asthma may be independent of neutrophils and instead mediated through macrophages [61]. For example, the m^6^A/METTL3/YTHDF3 axis has been shown to accelerate the degradation of Pentraxin 3 (PTX3) transcripts, thereby suppressing M2 macrophage activation. Since M2 macrophages promote Th2 response [62], this pathway could indirectly regulate allergic inflammation. Moreover, METTL3 suppresses PDGF-BB-induced airway smooth muscle cell (ASMC) proliferation and migration by downregulating the expression of TIMMDC1 [63] suggesting a potential regulatory role in airway remodeling associated with asthma.

However, the role of METTL3 is not exclusively protective. Some studies indicate that METTL3 may exacerbate allergic asthma by suppressing M2 macrophage activation through the PI3K/AKT and JAK/STAT6 pathways [64]. Conversely, METTL3 deficiency can mitigate asthma severity by negatively regulating the NF-κB pathway and thereby limiting M1 macrophage activation [65]. Additionally, METTL3 has been implicated in asthma progression through its involvement in DNA damage pathways, particularly via an exosomal lncRNA-mediated PAET-METTL3-YTHDF3-COX4I1 axis, which promotes DNA damage and worsens childhood asthma symptoms [66].

Interestingly, METTL3 exerts protective or pathogenic effects across asthma endotypes. It restrains Th2-driven allergic inflammation through m^6^A-dependent control of T cell and macrophage programs and may temper remodeling, yet it can aggravate neutrophilic disease via NETs-p300/H3K27ac-METTL3 routes that heighten epithelial damage and remodeling. These data support endotype and cell specific targeting of the METTL3 axis and careful consideration of disease stage when designing interventions.

Together, these findings highlight the context-dependent roles of METTL3 in different asthma subtypes and immune cell types. While it shows protective effects in Th2-dominant allergic asthma, METTL3 may also contribute to disease exacerbation under certain inflammatory or remodeling conditions, emphasizing the need for cell type– and disease endotype–specific therapeutic targeting. In asthma, METTL3 shapes both type-2 and neutrophilic endotypes through immune metabolic control. A NETs/NF-κB/METTL3 cascade likely amplifies epithelial injury in severe phenotypes. Future studies should consider stratifying by endotype and dominant cell of action to refine therapeutic targeting.

Clinical Implications (Asthma): Targeting the METTL3 axis in neutrophilic severe endotypes presents an opportunity for combination strategies incorporating anti-NET therapies and metabolic modulators.

### 2.2. METTL3 in Chronic Obstructive Pulmonary Disease

METTL3, a m^6^A “writer” responsible for RNA methylation, is a key regulator of gene ex pression and has been linked to the development of chronic obstructive pulmonary disease (COPD) [67], a progressive airway and pulmonary disease characterized by epithelial cell damage and extensive infiltration of neutrophils into lung tissue.

For example, in smoking-related COPD animal models, METTL3 expression is significantly upregulated in airway tissues and cigarette smoke extract (CSE)-treated human bronchial epithelial cells (HBECs). METTL3 promotes epithelial–mesenchymal transition (EMT) by inducing m^6^A methylation of SOCS3 mRNA, leading to reduced SOCS3 protein levels and activation of the STAT3/SNAI1 signaling pathway [68]. A 2025 study further demonstrated that METTL3 modifies OTUD1 mRNA via m^6^A methylation and facilitates its degradation through YTHDF2, resulting in enhanced pyroptosis and increased inflammatory cytokine release (IL-1β, IL-18) [69]. Collectively, these findings suggest that METTL3 contributes to COPD pathogenesis through the regulation of EMT, inflammatory responses, and autophagy, highlighting its potential as a novel therapeutic target in chronic airway diseases.

Ferroptosis is a programmed cell-death modality driven by iron-catalyzed lipid peroxidation and contributes to airway remodeling and emphysema. Emerging evidence indicates a critical role for ferroptosis in COPD, particularly in airway and alveolar epithelial cells, while METTL3 has emerged as a regulator of ferroptosis. One study identified that circSAV1 undergoes METTL3-mediated m^6^A methylation, which recruits YTHDF1 to enhance IREB2 translation, thereby initiating ferroptosis [70]. Furthermore, neutrophil extracellular traps (NETs) have been shown to induce alveolar epithelial cell ferroptosis via METTL3-mediated m^6^A modification [60], with the IGF2BP2 reader facilitating m^6^A regulation of HIF-1α, ultimately reprogramming mitochondrial metabolism. Ferroptotic injury in bronchial epithelial cells also promotes M2 macrophage polarization, thereby contributing to tissue remodeling and emphysematous changes in COPD [71].

Unlike asthma, the severity of COPD is strongly associated with NET burden. NETs are abundantly present in the sputum of COPD patients [72], and their levels increase during disease progression. COPD itself may promote excessive NET formation, largely mediated by upregulation of TLR4, a key sensor of pathogen-associated signals. Notably, METTL3 has been found to regulate neutrophil activation by modulating TLR4 signaling pathway through m^6^A-dependent mechanisms [73], further linking METTL3 to the amplification of neutrophilic inflammation and NET-driven epithelial damage in COPD.

In COPD, METTL3 integrates EMT and inflammatory signaling and intersects with ferroptosis and oxidative stress. Evidence points to SOCS3/STAT3 and EMT transcription factors as key downstream nodes. These features support combinations with metabolic or anti-NETs strategies, particularly during acute exacerbations.

Together, these studies suggest that METTL3 functions as a multifaceted regulator in COPD pathogenesis, influencing inflammatory signaling, cell death pathways, and immune cell polarization. These findings underscore the potential of targeting METTL3-mediated m^6^A methylation for therapeutic intervention in chronic inflammatory lung diseases such as COPD.

Clinical Implications (COPD): The EMT and inflammation nodes highlighted by our findings suggest that METTL3 modulation may be most effective when paired with antioxidant or STAT3-focused interventions, especially in the setting of acute exacerbations.

### 2.3. METTL3 in Lung Infection

Pulmonary infections, caused by viral, bacterial, or fungal pathogens, lead to inflammation of lung tissue and may result in clinical syndromes such as pneumonia, bronchitis, or tuberculosis. Cough, fever, difficulty breathing, and fatigue are among the most common symptoms. Treatment strategies vary depending on the pathogen and may involve antibiotics, antiviral or antifungal agents, and supportive care. The pulmonary surface is protected by a surfactant layer enriched with proteins such as surfactant protein A (SP-A) and surfactant protein D (SP-D), which play critical roles in preventing infection and modulating immune responses. Operating as effectors of the innate immune response, these proteins facilitate the clearance of pathogens and apoptotic cells by activating alveolar macrophages and dampening pulmonary inflammation [74,75].

Recent studies have identified METTL3 as a central epigenetic regulator in both viral and bacterial lung infections, including pneumonia, acute respiratory distress syndrome (ARDS), and sepsis-related inflammation. By modulating m^6^A methylation of immune and inflammatory transcripts, METTL3 alters the stability and translation of key mRNAs, thereby shaping the immune response and disease outcome. As such, it has been increasingly recognized as a promising therapeutic target in lung infections.

For example, LPS stimulation upregulates METTL3 expression in pulmonary endothelial cells, accompanied by the activation of NF-κB and NLRP3 pathways, leading to enhanced inflammation and endothelial barrier dysfunction [76]. In CLP-induced sepsis-associated lung injury, lactate-driven H3K18 lactylation increases METTL3 expression, which in turn stabilizes ACSL4 mRNA via m^6^A methylation and YTHDC1 recognition, thereby promoting ferroptosis in alveolar epithelial cells [77]. Moreover, in radiation-induced lung injury (RILI), METTL3 facilitates YY1 mRNA methylation, and its stability is enhanced by IGF2BP1, leading to fibroblast activation and pulmonary fibrosis, underscoring its critical role in radiation-associated lung damage [78].

METTL3 also plays a regulatory role in macrophage function, which is crucial in the con text of infection-driven lung inflammation [79,80]. In COVID-19, a viral infection caused by SARS-CoV-2, the formation of neutrophil extracellular traps (NETs) has been shown to worsen lung inflammation and vascular permeability. Excessive neutrophil activation contributes to the release of proteolytic enzymes, reactive oxygen species (ROS), and NETs, all of which compromise the integrity of the pulmonary microvasculature, particularly in influenza pneumonia and COVID-19 case [81,82,83,84]. Elevated levels of NETs, myeloperoxidase (MPO), and neutrophils have been observed in the pulmonary vessels of patients with COVID-19 [84], suggesting that targeting NETs may offer therapeutic potential. Notably, NETs have been found to induce METTL3 upregulation in alveolar epithelial cells through activation of the TLR9/MyD88/NF-κB pathway [85]. This mechanistic link further supports the notion that METTL3 may mediate inflammation and injury in viral lung infections.

METTL3 is emerging as a critical epigenetic regulator in various pulmonary infectious and inflammatory diseases. By modulating m^6^A RNA methylation in response to bacterial toxins, viral infections, sepsis, and radiation, METTL3 influences immune signaling, cell death, and tissue remodeling. These findings highlight its potential as a therapeutic target in conditions such as pneumonia, COVID-19, acute lung injury (ALI), ARDS, and radiation-induced lung damage.

In summary, METTL3 is a central epigenetic switch in infection-driven lung injury. Through reader-dependent m^6^A routing, it tunes NF-κB/NLRP3 signaling, metabolic stress, and ferroptosis, linking NETs-TLR9/MyD88 activation to epithelial damage and barrier failure across pneumonia/COVID-19/ALI-ARDS/RILI.

Clinical Implications (Lung infection): When epithelial pro-inflammatory signaling is predominant, early pharmacologic inhibition of METTL3 may offer the greatest therapeutic leverage. Incorporation of anti-NET strategies could interrupt the NET–NF-κB–METTL3 amplification circuit. In sepsis and ALI characterized by ACSL4-dependent ferroptotic injury, anti-ferroptosis agents warrant consideration. Employing reader-aware and cell-selective delivery platforms may help preserve endothelial barrier function.

## 3. METTL3 in Pulmonary Fibrosis

Pulmonary fibrosis (PF) represents a progressive interstitial lung disorder for which no curative therapy exists, and it is marked by aberrant extracellular matrix (ECM) accumulation, loss of alveolar elasticity, and chronic inflammation. A hallmark of PF is tissue hypoxia caused by persistent epithelial injury and inflammatory cell infiltration. Environmental pollutants, particularly particulate matter (PM2.5), have been recognized as important inducers of pulmonary fibrosis by triggering oxidative stress, ferroptosis, epithelial-mesenchymal transition (EMT), inflammatory cytokine release, and post-transcriptional gene regulation, including m^6^A RNA methylation modifications [86,87,88].

Evidence indicates that m^6^A modification participates in PM2.5-induced pulmonary fibrosis [89]. METTL3 has emerged as a key regulator of pulmonary-fibrosis pathogenesis. In models of radiation-induced lung injury, METTL3 stabilizes YY1 mRNA via an m^6^A-dependent mechanism, with IGF2BP1 recognizing and stabilizing the modified transcript, thereby promoting fibroblast activation and extracellular matrix (ECM) deposition [78]. Another study revealed that METTL3-mediated m^6^A modification of the long non-coding RNA lnc668 facilitates its phase separation–dependent export via YTHDC1, leading to PICALM upregulation and accelerated fibroblast-to-myofibroblast transition. In bleomycin-induced fibrosis, METTL3 also increases m^6^A levels on KCNH6 mRNA, enhancing its translation through YTHDF1, which further exacerbates fibroblast activation [90]. Additionally, METTL3 is broadly upregulated across various fibrotic lung diseases, regulating classic pro-fibrotic pathways such as TGF-β and COL1A1 [91]. Collectively, these findings demonstrate that METTL3 promotes pathological lung remodeling through multiple m^6^A-dependent mechanisms and represents a promising epigenetic therapeutic target in pulmonary fibrosis.

Following PM2.5 exposure, activation of molecular pathways such as TGF-β and fibro blast growth factors further amplifies EMT and fibrosis. METTL3 promotes fibrosis through multiple mechanisms. For example, it enhances the m^6^A methylation of CDH1 mRNA, regulated by miR-494-3p/YTHDF2 [92], thereby promoting EMT and fibroblast activation. Moreover, METTL3 expression is significantly increased in mice after PM2.5 exposure, where it promotes fibrosis by stabilizing the mRNAs of pro-fibrotic genes [93]. During TGF-β1 stimulation, METTL3 facilitates pri-miR-2 maturation, which suppresses PTEN and activates the NF-κB pathway in alveolar epithelial cells, thus aggravating fibrosis [94,95]. In contrast, inhibition of METTL3 alleviates PM2.5-induced lung injury by downregulating IL-24 expression through reduced m^6^A modification and YTHDF1 recruitment [96]. In addition to EMT and inflammation, ferroptosis, an iron-dependent form of regulated cell death, is increasingly recognized as a driver of PF. PM2.5 penetrates deep into alveolar epithelial cells, disrupts mitochondrial membrane potential, and induces oxidative imbalance, triggering ferroptosis [88]. NETs (neutrophil extracellular traps) further exacerbate alveolar epithelial ferroptosis by upregulating METTL3 via the IGF2BP2-dependent m^6^A modification pathway, enhancing glycolysis and suppressing oxidative phosphorylation [85]. Moreover, KCNH6, which encodes a potassium channel, has been linked to EMT and fibrosis. METTL3 promotes the YTHDF1-dependent translation of KCNH6, thereby accelerating EMT and myofibroblast proliferation, worsening idiopathic pulmonary fibrosis (IPF) [90].

Oxidative stress is another critical driver of PF [97], activating transcription factors such as Nuclear factor erythroid 2–related factor 2 (Nrf2). Nrf2 plays a protective role by inducing antioxidant and anti-inflammatory pathways. PM2.5-induced upregulation of METTL3 enhances Nrf2 translation via m^6^A methylation [98], with YTHDF1 and IGF2BP1 binding at specific mRNA sites (e.g., 1317, 1376, and 935), thereby reinforcing the antioxidant response and partially alleviating fibrosis [99]. Additionally, melatonin has been shown to protect against PM2.5-induced lung injury by activating Nrf2 and inhibiting ferroptosis in epithelial cells [86]. While METTL3’s role in pulmonary fibrosis has been increasingly elucidated, research on its function in other types of interstitial lung diseases (ILDs) remains limited. Given the significant health burden of ILDs, further studies are warranted. Collectively, these findings position METTL3 as a key epigenetic modulator in PM2.5-induced pulmonary fibrosis. Targeting METTL3-mediated m^6^A RNA modification and its downstream effectors may offer novel and promising therapeutic strategies for preventing or reversing fibrotic lung disease.

In pulmonary fibrosis, METTL3 drives fibroblast activation and ECM deposition via selective m^6^A programs, with the KCNH6-YTHDF1 axis linked to myofibroblast transition. These data support testing METTL3 modulation alongside approved anti-fibrotic agents in staged preclinical designs.

Clinical Implications (Pulmonary fibrosis): METTL3-targeted inhibition, coupled with established anti-fibrotic therapies, should be tested in rigorously staged preclinical models.

## 4. METTL3 in Pulmonary Vascular Disease

Pulmonary vascular disease (PVD) refers to a group of disorders affecting the pulmonary vasculature, often leading to complications such as pulmonary hypertension (PH), reduced oxygenation, and ultimately right heart failure. These conditions carry a substantial burden of morbidity and mortality. Recent investigations have emphasized METTL3, as a critical epigenetic regulator in the pathogenesis of PVD.

METTL3 contributes to pulmonary vascular remodeling by promoting pulmonary artery smooth muscle cell (PASMC) proliferation through m^6^A modification of target genes such as RBPJ and GLUT4 [100], At the same time, METTL3 exerts protective effects on pulmonary vascular endothelial cells by stabilizing transcripts like PTEN [101] and KLF2 mRNAs [102], which suppress inflammation and endothelial-to-mesenchymal transition (EndMT). Under pathological conditions such as hypoxia or elevated pulmonary pressure, downregulation of METTL3 may exacerbate disease progression, whereas under physiological conditions, it contributes to the maintenance of endothelial homeostasis.

Although METTL3 has been extensively studied in cancer biology and stem cell differentiation [103], its role in pulmonary hypertension (PH) and pulmonary arterial hypertension (PAH) has recently gained attention. Dysregulation of METTL3 and aberrant m^6^A methylation are increasingly recognized as contributors to pathological cell proliferation, apoptosis resistance, and vascular remodeling, hallmarks of PH pathogenesis [104,105,106].

A central aspect of METTL3’s function in PVD is its regulation of vascular endothelial cells and smooth muscle cells [107,108]. Under hypoxic conditions, METTL3 expression is upregulated, enhancing m^6^A modification of mRNAs that encode pro-proliferative and anti-apoptotic proteins. This promotes PASMC proliferation and contributes to EndMT in pulmonary arterial endothelial cells (PAECs) [102,109]. One well-studied example is HIF-1α, a master transcription factor activated by hypoxia [110,111]. METTL3-mediated m^6^A methylation stabilizes HIF-1α mRNA, enhancing its translation and leading to increased protein expression under hypoxic stress [112]. This upregulation contributes to angiogenesis, metabolic reprogramming, and vascular remodeling. In addition, METTL3 also influences the expression of VEGF [113], further driving fibrotic and vascular changes. Adipose-derived stem cells (ADSCs), a type of mesenchymal stem cell, possess the capacity to differentiate into vascular smooth muscle cells (VSMCs). Hypoxic stress accelerates this differentiation. METTL3 plays a critical role in this process, as METTL3 knockdown reduces hypoxia-induced VSMC differentiation from ADSCs, indicating that METTL3 mediates hypoxia-driven lineage commitment in pulmonary vascular disease [107].

Moreover, METTL3 regulates the expression and function of non-coding RNAs, including microRNAs and long non-coding RNAs, through m^6^A modifications. These epigenetic changes impact transcript stability, splicing, and translation, thereby influencing vascular homeostasis. For example, m^6^A-modified LINC02038 has been proposed to act as a miRNA sponge, sequestering miR-552-5p [114], which targets pro-proliferative and anti-apoptotic genes, ultimately promoting vascular cell proliferation [115]. Importantly, studies in PH animal models have shown that METTL3 and YTHDF1 expression levels are elevated in whole lung tissues of PH rats. Pharmacological or genetic modulation of METTL3 can improve disease phenotypes, reduce vascular remodeling, and restore pulmonary hemodynamics [116]. These findings suggest that targeting METTL3 or the broader m^6^A epitranscriptomic machinery could represent a novel therapeutic strategy for treating PVD.

In conclusion, METTL3 functions as a versatile regulator in the development of pulmonary vascular disease by regulating mRNA stability, non-coding RNA activity, cell proliferation, and differentiation pathways in response to environmental and pathological stimuli. A deeper understanding of METTL3-mediated m^6^A dynamics in pulmonary vascular cells will pave the way for precision epigenetic therapies aimed at reversing or halting disease progression in PVD.

Across pulmonary vascular disease, METTL3 exerts opposing effects in smooth muscle versus endothelium, underscoring the need for targeted delivery. Hypoxia/HIF-1α signaling is a convergent pathway. Precision dosing and vascular-cell-specific targeting will be critical for safe and effective translation.

Clinical Implications (Pulmonary vascular disease): Cell-selective delivery approaches should be emphasized to achieve an optimal balance between preserving endothelial function and regulating smooth muscle remodeling.

## 5. METTL3 in Acute Respiratory Disease Syndrome

Acute lung injury (ALI) and its severe manifestation, acute respiratory distress syndrome(ARDS), are critical respiratory conditions characterized by diffuse alveolar damage, inflammatory cell infiltration, and loss of alveolar–capillary membrane integrity [117]. These syndromes can result from both direct (e.g., infection, aspiration) and indirect (e.g., sepsis, trauma) pulmonary insults [117]. Despite progress in intensive care, ARDS mortality remains high (40–60%) [118], underscoring the urgent need to understand the underlying mechanisms and develop targeted therapeutic interventions.

In recent years, m^6^A RNA modification, largely driven by METTL3, has been recognized as a pivotal regulatory pathway in ALI/ARDS and other pulmonary diseases [119]. Studies have found that in ALI, both the level of m^6^A methylation and the expression of METTL3 increase in ALI/ARDS [120]. METTL3-mediated m^6^A methylation is enriched in the mRNA of inflammatory regulatory genes, highly suggesting a central role of METTL3 in inflammatory regulation as a hub controller during ARDS [120]. However, METTL3 exhibits cell type–specific expression patterns within pulmonary tissue: its levels are reduced in alveolar epithelial cells (AECII) and in HUVECs, remains stable in macrophages, but increases in neutrophils [121]. These discrepancies reflect the complex regulatory balance between methyltransferases and demethylases in lung injury and repair, emphasizing the importance of context-dependent analysis of METTL3’s function.

### 5.1. Dual Functions of METTL3 in ALI/ARDS

METTL3 demonstrates context-dependent dual roles in ALI/ARDS, acting as both a pro-inflammatory factor and a regulator of repair, depending on the cell type and disease stage. In alveolar epithelial cells, METTL3 installs m^6^A on PTEN transcripts and enhances their stability, which augments inflammatory signaling and constrains cell proliferation. Consistent with this, METTL3 knockdown reduces inflammation and promotes type II alveolar epithelial cell (AECII) proliferation [121] and surfactant related recovery, thereby mitigating lung injury. By contrast, in sepsis-induced ALI, METTL3 appears protective: activation of the Trim59-NF-κB axis [122] helps curb inflammation and maintain endothelial barrier integrity. These findings highlight the context-dependent roles of METTL3 in lung pathology. Meanwhile, METTL3 also interfaces with metabolic and cell-death programs.

Collectively, METTL3 has dual potential: harmful in some contexts, beneficial in others. Management should be stratified by endotype, cell context, and timing. Using inhibition in epithelial-dominant, pro-inflammatory states while maintaining METTL3 activity when endothelial barrier support and resolution are foremost.

### 5.2. Role of METTL3 in Ferroptosis and Cell Death Pathways

Ferroptosis, an apoptosis-independent, iron-dependent death program characterized by lipid peroxidation, is implicated in ALI/ARDS pathogenesis. In sepsis-associated ALI, NETs induce METTL3 expression, which in turn promotes ferroptosis in alveolar epithelial cells. Silencing METTL3 reduces m^6^A modification levels and ferroptotic activity, leading to significant improvement in lung injury in both in vitro and in vivo models [85]. These observations imply that targeting METTL3 inhibitors may serve as a potential therapeutic approach for sepsis-related ALI/ARDS.

### 5.3. Regulation of Autophagy and Pyroptosis

METTL3 also modulates autophagy and pyroptosis, two key pathways influencing cell. survival and inflammatory responses in ALI/ARDS. In alveolar epithelial cells, m^6^A modification of Sirt1 transcripts mediated by METTL3 disrupts autophagic flux. METTL3 knockdown stabilizes Sirt1 expression, enhancing autophagy and reducing lung injury [123]. In macrophages, TTC4 inhibits pyroptosis by suppressing mitochondrial damage. METTL3 downregulates TTC4 expression via the HSP70/ROS/NLRP3 signaling pathway, thereby promoting macrophage pyroptosis and worsening lung inflammation [124]. Additionally, METTL3 protects vascular endothelial cells by regulating Trim59, which contributes to barrier integrity and suppression of excessive inflammatory responses [122]. Therefore, the role of m^6^A/METTL3 in regulating cellular responses to intrinsic and extrinsic stressors requires further investigation.

### 5.4. Conclusions and Future Directions

METTL3 plays a versatile and cell-specific role in the pathogenesis of ALI/ARDS. It regulates diverse processes including inflammation, cell death, barrier function, and tissue repair through m^6^A-dependent modulation of coding and non-coding RNAs. Its dual nature, as both a pro-inflammatory factor and cellular protector, suggests that METTL3-based therapies must be precisely tailored to disease stage and target cell type. Further elucidation of METTL3’s interactions with its downstream targets and signaling pathways in specific lung cell populations will be essential to develop effective and personalized therapeutic strategies for ALI/ARDS and related respiratory disorders.

ALI/ARDS exhibits context dependent outcomes of METTL3 modulation, reflecting strong cell and time-specific effects. Crosstalk with ferroptosis, autophagy, and pyroptosis appears central to barrier failure.

Clinical Implications (ALI/ARDS): Given the narrow therapeutic window in ALI/ARDS, early intervention integrating anti-ferroptosis and anti-NET approaches with METTL3 inhibition may offer maximal protection of epithelial–endothelial barrier integrity.

## 6. METTL3 in Lung Cancer

METTL3 has been repeatedly implicated in non-small cell lung cancer (NSCLC), where it can promote oncogenic programs through m^6^A-dependent translational enhancement and mRNA stabilization. In several NSCLC models, METTL3 cooperates with translation initiation machinery (e.g., eIF3h) to increase the translational efficiency of oncogenic transcripts, thereby sustaining proliferation, invasion, and therapy tolerance. In parallel, reader proteins, notably IGF2BP family members can stabilize m^6^A-tagged targets such as MYC and HIF-1α, reinforcing glycolytic reprogramming, hypoxic signaling, and an immunosuppressive tumor microenvironment.

Importantly, context-dependent tumor-suppressive effects of METTL3 have also been reported, reflecting cell type, isoform, and microenvironmental dependencies. Such bidirectionality echoes observations in non-malignant lung diseases and underscores the need for patient stratification by cellular context, m^6^A site–level biology, and reader usage.

Translational perspective: Given these dual roles, therapeutic strategies may include: (1) direct METTL3 inhibition in tumors where oncogenic outputs dominate; (2) reader-targeted approaches (e.g., modulating IGF2BP–target interactions) to decouple malignant stabilization from homeostatic mRNA control; and (3) combination regimens that pair METTL3-axis modulation with anti-glycolytic, anti-hypoxia, or immune-checkpoint therapies. Prospective studies integrating single-cell, spatial transcriptomics and m^6^A profiling will be crucial to define responders, timing, and on-target pharmacodynamic markers.

Clinical Implications (Lung cancer): METTL3 inhibition is best suited for tumors demonstrating METTL3-dependent, eIF3h-facilitated translational activation and IGF2BP-mediated stabilization of MYC or HIF-1α pathways. In tumors lacking this axis, reader-directed interventions may provide superior therapeutic leverage. Rational combinations with anti-glycolytic or anti-hypoxia agents, as well as immune-checkpoint blockade, should be tailored to tumor biology. Biomarker-driven guidance, spanning m^6^A site and reader occupancy, HIF-1α/glycolysis signatures, and TME characteristics, is essential for patient stratification.

## 7. Common Mechanistic Themes of METTL3 in Lung DISEASES

**Figure 2:**
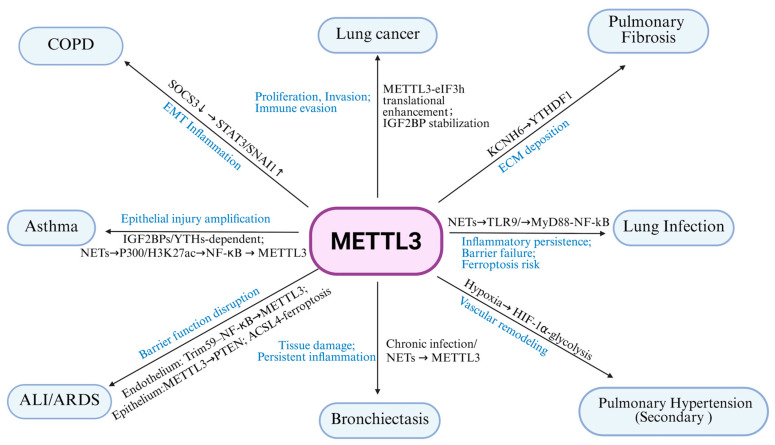
MTLLE3 related pathway and outcomes.

In the lung diseases discussed above, several recurrent patterns of METTL3 action can be observed (Figure 2 and Table 1). First, METTL3 often acts as a key responder to injurious stimuli, including cigarette smoke, PM2.5, NETs, hypoxia, LPS and sepsis-related mediators. These stimuli tend to converge on signaling pathways such as ERK, NF-κB and HIF-1α, as well as epigenetic regulators including p300 mediated H3K27 acetylation or histone lactylation. These upstream signals generally increase METTL3 expression, thereby enhancing m^6^A modification on selected transcripts and creating positive feedback loops linking inflammation, metabolic stress and tissue injury.

Second, METTL3 mediated m^6^A regulation repeatedly converges on a set of downstream axes that are shared across diseases. In airway and alveolar epithelium, METTL3 targets SOCS3/STAT3, ACSL4, KCNH6, PTEN and others to promote epithelial–mesenchymal transition, ferroptosis and barrier disruption, thereby tightly coupling inflammatory signaling to structural remodeling in COPD, pulmonary fibrosis and ALI/ARDS. In immune cells, METTL3 modulates Th2 differentiation, macrophage polarization, neutrophil activation and NET formation, thereby linking m^6^A modification to distinct inflammatory endotypes in asthma and infection related lung injury. In the pulmonary vasculature and in lung cancer, METTL3 stabilizes and promotes translation of HIF-1α, glycolytic enzymes and multiple growth factor pathways, supporting hypoxic adaptation, metabolic reprogramming and proliferative remodeling of smooth muscle cells, endothelial cells and tumor cells.

Third, the same METTL3-m^6^A pathway can exert either protective or pathogenic effects depending on cell type and disease stage. For example, in sepsis associated ALI, METTL3 may help maintain endothelial barrier integrity while exacerbating ferroptosis in epithelial cells. In allergic asthma, METTL3 may restrain Th2 responses but amplify tissue injury in severe, neutrophil and NET dominant subtypes. Likewise, METTL3 can drive fibrotic remodeling yet, by activating Nrf2 linked antioxidant programs, partially alleviate oxidative stress in PM2.5 induced lung injury. These apparently paradoxical actions are further shaped by the usage of different m^6^A readers (such as IGF2BP family members versus YTHDF proteins) and by crosstalk with other epigenetic marks.

Taken together, these shared mechanisms indicate that METTL3 is a central post transcriptional epigenetic hub that integrates environmental insults, immune signaling, metabolic reprogramming and cell death pathways in the lung. At the same time, they suggest that attempts to therapeutically target METTL3 must carefully consider disease stage, predominant cell types and the m^6^A reader context, and should be combined with strategies that modulate NET burden, ferroptosis and fibrotic or vascular remodeling to achieve safer and more effective interventions.

## 8. Clinical Implications of METTL3/m^6^A-Based Therapy

Clinical trials targeting METTL3 are significant in cancer therapy due to the role of METTL3 in RNA methylation and gene expression regulation (Table 2). These trials focus on evaluating the safety, pharmacokinetics, and anti-tumor efficacy of METTL3 inhibitors. Initial results show promise in enhancing immune responses and activating related pathways, which may improve treatment outcomes for advanced malignancies and offer new therapeutic strategies when combined with existing treatments such as checkpoint inhibitors.

Non-oncology translational prospects

ALI/ARDS: Early METTL3 inhibition combined with anti-NETs or anti-ferroptotic agents may stabilize epithelial/endothelial barriers; pharmacodynamic readouts should track barrier repair and m^6^A-site signatures.

Pulmonary fibrosis: Targeting METTL3-dependent myofibroblast programs (e.g., KCNH6–YTHDF1) may synergize with approved anti-fibrotics; staged designs can separate injury and remodeling phases.

Infection-driven lung injury: Reader-aware approaches could decouple host defense from persistent inflammation by modulating IGF2BP–target interactions.

Cross-cutting: Cell-targeted delivery, time-resolved dosing, and biomarker-guided stratification (cell/site/reader usage) are essential to increase the therapeutic index.

## 9. Conclusions

As a core methyltransferase in the m^6^A modification pathway, METTL3 plays a complex role in the pathogenesis of lung diseases (Summarized in Table 1 and Figure 2). By modulating m^6^A methylation in various lung cell types, METTL3 influences a range of biological processes, including inflammation, programmed cell death, ferroptosis, autophagy, and necroptosis. In diseases such as ALI/ARDS, pulmonary fibrosis, and lung infections, where effective therapeutic options remain limited, targeting METTL3 and the m^6^A pathway may offer novel treatment avenues.

Future studies should continue to investigate the cell-type–specific and disease-context–dependent roles of METTL3 across different lung disease models. A deeper understanding of how METTL3 contributes to pulmonary pathology, and how its activity can be modulated pharmacologically or genetically, will provide critical insights into m^6^A-targeted therapies. Ultimately, the integration of advanced m^6^A detection technologies with mechanistic research on METTL3 holds great promise for innovative therapeutic development in respiratory medicine.

## 10. Limitations and Challenges

Despite compelling preclinical data, several hurdles complicate the clinical translation of METTL3-centered strategies. (1) Cell and temporal specificity: The effect of METTL3 perturbation frequently depends on the disease stage and cellular context (epithelial; endothelial; immune lineages), with even opposite outcomes reported across settings. (2) Delivery and on-targeting: Systemic inhibition may affect hematopoiesis and neural tissues; tissue or cell targeted delivery and temporal control are therefore desirable. (3) Network redundancy: Compensation through ALKBH5/FTO and reader proteins (YTH/IGF2BP families) can blunt single node interventions. (4) Biomarker-guided stratification: Patient selection likely requires m^6^A site level and reader usage information rather than bulk METTL3 expression. (5) Combination design: Optimal pairing with anti-fibrotic, anti-inflammatory, anti-NETs, anti-ferroptotic, or metabolic modulators will require dose–time–cell triangulation. Addressing these challenges will reduce on target liabilities and increase the probability of clinical benefit.

## 11. Future Directions

(1) Context-specific METTL3 circuits: A major challenge is to precisely define how METTL3-dependent m^6^A programs are wired in different cell types and disease stages. Future work should combine single-cell and spatial transcriptomics with m^6^A profiling to resolve cell–site–reader triplets(writer-reader-target) and track their temporal switching during disease progression, especially in complex mixed endotypes such as severe asthma, COPD with frequent exacerbations, and sepsis-related ALI/ARDS. Such maps will be essential to identify tissue-specific or disease-phase-specific m^6^A regulations that are safe to target.
(2) Upstream regulation and PTMs of METTL3: It remains unclear how environmental cues (e.g., PM2.5, NETs, hypoxia, LPS) are integrated through post translational modifications of METTL3 such as phosphorylation and ubiquitination, and how these modifications differ between protective and detrimental contexts. Dissecting these signaling nodes may reveal opportunities to indirectly tune METTL3 activity, rather than globally blocking the enzyme.
(3) Rational combinations: Stage-specific combinations (e.g., METTL3 inhibition with anti-fibrotics, anti-NETs/anti-ferroptosis, or metabolic agents) need to be evaluated, guided by pharmacodynamic biomarkers and functional assays of barrier repair.
(4) Epigenetic crosstalk: Map hierarchies between m^6^A and other epitranscriptomic/epigenetic marks need to be determined (e.g., m^5^C, histone acetylation/methylation) to uncover cooperative or compensatory modules amenable to co-targeting.
(5) Translational tools and biomarkers: Finally, there is a need for more selective METTL3 inhibitors, reader specific modulators, and location targeted m^6^A editing tools suitable for in vivo use, as well as robust clinical assays for METTL3 expression, m^6^A load, and reader patterns in patient samples. Integrating these biomarkers into early-phase trials in pulmonary hypertension, fibrotic ILD, or severe asthma would be an important step toward precision epitranscriptomic medicine in lung disease.

## Figures and Tables

**Table 1:** MTLLE3-related pathway and outcomes in pulmonary diseases.

Disease	METTL3-Linked Pathway	Principal Cell	Pathophysiology	Translational Angle
COPD	SOCS3↓ → STAT3/SNAI1↑	Bronchial epithelium	EMT; Inflammation	METTL3 inhibitor + antioxidant/STAT3-modulating agents
Pulmonary fibrosis	KCNH6-YTHDF1	Fibroblast → myofibroblast activation	ECM deposition	METTL3 inhibitor +anti-fibrotic
ALI/ARDS	Epithelium: METTL3→PTEN↑; ACSL4-ferroptosis; Endothelium: Trim59–NF-κB→METTL3	AEC II; Endothelium	Epithelium: Barrier failure Endothelium: Barrier protection	Early METTL3 inhibition + anti-NETs/anti-ferroptosis
Asthma	NETs→P300/H3K27ac→NF-κB → METTL3; reader-dependent routing (IGF2BPs/YTHs)	Airway epithelium; neutrophils	Epithelial injury amplification	METTL3-axis modulation + anti-NETs/metabolic agents
Bronchiectasis	Chronic infection/NETs → METTL3	Airway epithelium; neutrophils	Persistent inflammation; Tissue damage	Anti-NETs + METTL3-axis modulation (context-dependent)
Secondary pulmonary hypertension	Hypoxia → HIF-1α-glycolysis; METTL3-reader programs	Endothelium; smooth muscle	Vascular remodeling	Cell-targeted delivery; reader-aware interventions
Infection-driven lung injury	TLR-NF-κB-METTL3; IGF2BP-mediated stabilization	Epithelium; Endothelium; Macrophages	Inflammatory persistence; Ferroptosis risk; Barrier failure	Reader-aware approaches; anti-ferroptosis + METTL3
Lung cancer (NSCLC)	METTL3-eIF3h translational enhancement; IGF2BP stabilization (MYC, HIF-1α)	Cancer cells; TME	Proliferation, invasion; Immune evasion	METTL3 inhibition; reader-targeting; +anti-glycolysis

**Table 2:** Therapeutic Development targeting m^6^A RNA modifications.

Intervention	Target	Indication	Trial Start Date	Company	Status (Start Date)	Identifier
**STM-2457/STC-15**	METTL3	AML	23 November 2022	STORM Therapeutics	Phase 1 (2022)	NCT05584111
**Unidentified**	METTL3	AML, NSCLC	Unknown	Accent Therapeutics	Phase 1	N/A
**Unidentified**	METTL3	AML	Unknown	Gotham Therapeutics	Phase 1	N/A
**METTL3 inhibitor**	METTL3	AML (select sub-types), solid tumours	Unknown	Ipsen/Accent Therapeutics	IND-enabling (pre-clin. 2021)	N/A
**EP102**	METTL3	AML	Unknown	EPICS Therapeutics	pre-clinical	N/A
**Unidentified**	METTL3	AML	2021	Ipsen and Accent Therapeutics	pre-clinical	N/A
**dCas13-METTL3 editor**	METTL3	Ex vivo MYC, HBB	Unknown	Beam Therapeutics × StemiRNA	In vitro validation (2021)	N/A
**WD6305**	METTL3-METTL14 complex	AML	Unknown	Shanghai Institute of Materia Medica	pre-clinical (2024)	N/A
**siMETTL3/siFTO(LNP)**	METTL3/FTO	Liver, cancer models	Unknown	Alnylam/Arrowhead	Pre-clinical tox complete (2024)	N/A
**FB23-2**	FTO	AML, IDH-mut glioma (pre-clin models)	Unknown	CuraTe Therapeutic	Pre-clinical (2019)	N/A
**CS1/CS2**	FTO	AML, glioma	Unknown	CuraTe Therapeutic	Tool use only (2022)	N/A
**FTO-PROTAC (QP73)**	FTO	AML (pre-clin models)	Unknown	ProtaGene Bio	Proof-of-concept (2024)	N/A
**ALK-04**	ALKBH5	Melanoma, RCC + anti-PD-1	Unknown	Epiprev Biotech	Pre-clinical (2020)	N/A
**IOX1 (broad 2-OG dioxygenase blocker)**	ALKBH5	GBM (combo with checkpoint inhibitor)	Unknown	Tool compound (various labs)	Tool use only (2011)	N/A
**CK-75**	YTHDF2	B-cell malignancies, solid-tumour models	Unknown	Discovery stage (Phenyl-pyrazole series)	Pre-clinical (2024)	N/A

Note: STM-2457 is a selective METTL3 inhibitor developed by STORM Therapeutics, currently in Phase 1 trials for AML; STC-15 is another potent METTL3 inhibitor with activity against chemotherapy-resistant leukemia, and is the first RNA methyltransferase inhibitor to enter clinical development; WD6305 and EP102 are under pre-clinical investigation for AML; Additional METTL3-targeting compounds are being developed by Accent, Gotham, and Ipsen, mostly in pre-clinical or IND-enabling stages; Several FTO inhibitors (e.g., FB23-2, CS1/CS2, FTO-PROTAC) are in tool-use or proof-of-concept phases for AML and glioma; ALK-04 and IOX1 target ALKBH5 for melanoma, RCC, and other cancers; CK-75, a YTHDF2-targeting agent, is in discovery/pre-clinical stages for B-cell and solid tumors; siMETTL3/siFTO (LNP) therapies (Alnylam/Arrowhead) have completed pre-clinical toxicology in liver and cancer models; A dCas13-METTL3 editor (Beam/StemiRNA) is being evaluated in ex vivo gene editing. Abbreviations: AML: Acute myeloid leukemia; METTL3: methyltransferase-Like 3; PH: Pulmonary Hypertension; RCC: Rental Cell Carcinoma; GBM: Glioblastoma; FTO: Fat Mass and Obesity-Associated Protein; ALKBH5: AlkB Homolog 5; YTHDF2: YTH N6-Methyladenosine RNA Binding Protein 2; LNP: Lipid Nanoparticle.

## Data Availability

No new data were created or analyzed in this study. Data sharing is not applicable to this article.
